# The impact of benzodiazepine use in patients enrolled in opioid agonist therapy in Northern and rural Ontario

**DOI:** 10.1186/s12954-017-0134-5

**Published:** 2017-01-26

**Authors:** Alexandra M. Franklyn, Joseph K. Eibl, Graham Gauthier, David Pellegrini, Nancy K. Lightfoot, David C. Marsh

**Affiliations:** 1 Northern Ontario School of Medicine, Sudbury, ON P3E 2C6 Canada; 2 School of Rural and Northern Health, Laurentian University, 935 Ramsey Lake Rd., Sudbury, ON P3E 2C6 Canada; 3 Canadian Addiction Treatment Centers, 13291 Yonge St., Ste. 403, Richmond Hill, Ontario L4E 4L6 Canada

**Keywords:** Opioid agonist therapy, Treatment retention, Northern Ontario, Benzodiazepines, Concurrent drug use

## Abstract

**Background:**

Benzodiazepine use is common among patients in opioid agonist therapy; this puts patients at an increased risk of overdose and death. In this study, we examine the impact of baseline and ongoing benzodiazepine use, and whether patients are more likely to terminate treatment with increasing proportion of benzodiazepine positive urine samples. We also study whether benzodiazepine use differs by geographic location.

**Methods:**

We conducted a retrospective cohort study using anonymized electronic medical records from 58 clinics offering opioid agonist therapy in Ontario. One-year treatment retention was the primary outcome of interest and was measured for patients who did and did not have a benzodiazepine positive urine sample in their first month of treatment, and as a function of the proportion of benzodiazepine-positive urine samples throughout treatment. Cox proportional hazard model was used to characterize one-year retention.

**Results:**

Our cohort consisted of 3850 patients, with the average retention rate of 43.4%. Baseline benzodiazepine users had a retention rate of 39.9% and non-users had a retention rate of 44%. Patients who were benzodiazepine negative on admission benefited from an increased median days retained of 265 vs. 215 days. Patients with more than 75% of urines positive for benzodiazepines were 175% more likely to drop out of treatment than those patients with little or no benzodiazepine use.

**Conclusions:**

Baseline benzodiazepine use is predictive of decreased retention. Patients who have a higher proportion of benzodiazepine-positive urine samples are more likely to drop out of treatment compared to those who have little or no benzodiazepine detection in their urine.

## Background

Opioids are one of the most frequently prescribed medications in Canada, and the misuse of prescription opioids is becoming increasingly more prevalent [1]. In Ontario, the number of opioid-related deaths increased by an alarming 242% between 1991 and 2010 [2]. This problem is particularly severe in Northern Ontario, which is home to the highest rates of both opioid prescribing and opioid-related death within the province [3].

In a retrospective study on opioid-related deaths in Ontario, 59.5% of deaths involved benzodiazepines (BZD), a family of non-opioid central nervous system depressants [1]. The short-term use of BZD is clinically indicated in patients who suffer from anxiety, seizures, or acute alcohol withdrawal. BZD are considered to be high risk for dependence [4], especially when used long-term [5]. Long-term BZD dependence can cause serious harm, including impaired sleep, decreased mood, and a decline in cognitive function [6].

BZD are often prescribed to patients who are receiving opioid agonist therapy (OAT) [7]. In a cross sectional survey of patients in OAT, two-thirds of patients reported concurrent BZD use [8]. Another cross-sectional study of 170 patients receiving OAT found that 24% met the criteria for BZD dependence, according to the diagnostic statistical manual of mental disorders-IV (DSM-IV) [9]. The use of BZD during OAT puts patients at a greater risk of overdose and death [10]. An Alabama review of methadone fatalities that involved other drugs found that BZD were detected in 59% of deaths [11]. Additionally, patients who are prescribed methadone and use BZD on an ongoing basis are more likely to continue polydrug use during treatment, including cocaine and other opioids [10]. While previous studies reveal mixed findings about whether ongoing BZD use negatively affects treatment retention, BZD use during treatment has been correlated with a more complex clinical course [10, 12, 13]. Additionally, BZD misuse is correlated with negative patient outcomes, such as unemployment, involvement in criminal activity, and psychological distress [10].

A review of the literature reveals that most BZD prescribing is in agreement with clinical guidelines; however, there does exist some prescribing that is contrary to clinical guidelines [14]. A questionnaire answered by 66 internationally recognized experts on pharmacotherapy [15] suggested that the risks of BZD are overstated and revealed support of long-term BZD use in patients suffering from anxiety, despite the lack of evidence for effectiveness of long-term BZD use [16]. A qualitative study of 35 general practitioners revealed that physicians are often cautious when initiating BZD use in their patients; however, they view the prescribing of BZD as “the lesser evil” compared to the patient’s psychosocial problems [17].

Along with concurrent drug use, geography is another important factor when studying OAT retention rates. In this study, we focus on differences in BZD use and retention between Northern and Southern Ontario, as well as rural and urban Ontario. An important difference between the North and South is the population density. Northern Ontario has approximately 10% of the population in about 90% of the geographic area. It is for this reason that patients often have to travel large distances to access OAT services and pharmacies to dispense their medication [18]. Despite facing a variety of barriers when accessing care, patients in the North experience higher treatment retention rates than patients in Southern Ontario [18]. Further research must be done to better understand why this occurs, including whether patterns of current drug use—such as BZD use—plays a role.

While the potential risks of BZD use during OAT are clear, it is not yet clear whether abstaining from BZD during OAT is beneficial for patients who suffer from co-occurring mental health disorders, where BZD may be clinically indicated. In this study, we evaluate treatment retention for patients who use BZD and those who do not. Further, we also evaluate whether BZD use differs by geographic location.

## Methods

### Cohort definition

We conducted a retrospective cohort study of patients initiating OAT for the first time between January 1st, 2011 and June 17th, 2012 in the Province of Ontario. We defined first time OAT as no previous history of methadone or buprenorphine/naloxone use in the network of clinics studied, based on review of records dating back to 1999. Patients started on either methadone or Suboxone (buprenorphine/naloxone), which were the only medications approved for opioid dependence in Canada at the time of the study. Patients were allowed to transition between these two medications over the course of treatment. Patients were at least 15 years or older (patients <18 years of age accounted for <1% of cohort), and were residents of Ontario. All patients were followed from their date of OAT initiation to the date of drug discontinuation (patient did not receive a methadone or buprenorphine dose for 30 consecutive days), or end of the study period (June 2013).

### Data sources

The dataset used for this study was derived from anonymized electronic medical records from a group of 58 addiction treatment centers across the Province of Ontario–the Ontario Addiction Treatment Centers (OATC). Prior to data analysis, personal identifiers were replaced with an encrypted unique identifier.

### BZD use

Patients were categorized by baseline BZD use (defined as having any BZD-positive urine samples in their first month of treatment) and by ongoing BZD use (determined by the proportion of BZD-positive urine samples throughout treatment). Urine toxicology screening was performed one to two times per week on all patients throughout their time in care via an enzyme immunoassay, which has the ability to detect BZD in the urine [19]. However, this test is unable to differentiate between different BZDs, which include (but are not limited to): diazepam, clonazepam, and lorazepam. The majority of urine toxicology screens reported were conducted using an antibody reactive to diazepam (and related compounds); therefore, the use of clonazepam may be underestimated. The detection period and sensitivity differs for each BZD, ranging from a few hours to a few days [19].

### Definition of treatment retention

Unless treatment was terminated, all patients were followed for at least one year to a maximum follow-up date of June 17th, 2013. Continuous OAT was assessed on the basis of not having a period of 30 or more consecutive days without a dose of medication. We defined a patient as having been retained in treatment if they completed at least one year of continuous and uninterrupted OAT. There is considerable evidence to suggest that one year treatment retention is correlated with a variety of positive health outcomes, including reduced drug use, relapse, hospitalization, and illegal activity [20, 21]. In the event that a patient transitioned to a non-OATC clinic, was incarcerated, hospitalized, or was otherwise prevented from refilling their prescription, it is possible for type 1 error to occur.

### Statistical analysis

**Fig. 1:**
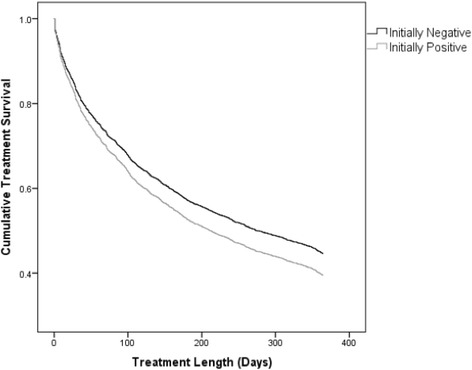
Treatment retention by baseline BZD use. A Cox proportional hazard analysis was used to characterize the time to treatment discontinuation between the patient groups. Log-rank comparison of these curves yielded a chi-square value of 2.883 with a non-significant *p* value of 0.0895. Baseline BZD users were 14.9% more likely to drop out of treatment than baseline non-users [_a_HR = 1.15(95% CI 1.02–1.29)]

Descriptive statistics were summarized for baseline characteristics of patients, and standardized differences were used to compare patients in the various BZD use groups (Table 1). Baseline characteristics included: percentage of patients that were male/female, Northern/Southern, and rural/urban, median age, median peak methadone dose, median days retained, the percentage of BZD-positive urine samples, and the one-year retention rate. For the purpose of this study, only a patient’s first treatment episode was considered. For the primary analysis (risk of treatment drop out), a Cox proportional hazard analysis was used to characterize the time to treatment discontinuation (Fig. 1). The relative likelihood of treatment termination between the BZD positive and negative patient groups was calculated from hazard ratios, adjusting for the impact of age, gender, Northern and rural location. Cox proportional hazard analysis and log-rank test were performed using SPSS 24.

**Table 1:** Characteristics of baseline BZD users and non-users

		Initially negative (*n* = 3288, 85.4%)	Initially positive (*n* = 562, 14.6%)
Male/female		1975 (60.1%)/1313 (39.9%)	316 (56.2%)/246 (43.8%)
North/South		1239 (37.7%)/2048 (62.3%)	166 (29.5%)/396 (70.5%)
Urban/rural		2753 (83.8%)/543 (16.2%)	499 (88.8%)/63 (11.2%)
Median age (*Q*_1_, *Q*_3_; SD)		30.8 (25.3, 39.4; SD = 10.2)	34.3 (28.1, 45.3; SD = 10.9)
Median peak methadone dose (*Q*_1_, *Q*_3_; SD)		75 (50, 100; SD = 33)	85 (55, 115; SD = 36)
Median peak suboxone dose ( *Q*_1_, *Q*_3_; SD)		8 (8, 16; SD = 7)	12 (8, 20; SD = 8)
Median days retained (*Q*_1_, *Q*_3_; SD)		265 (56, 526; SD = 272)	215 (53, 519; SD = 270)
Median percent positive results (*Q*_1_, *Q*_3_; SD)		0.0 (0.0, 0.0; SD = 4.9)	21.4 (6.8, 55.1; SD = 32.1)
Percent positive results	[0, 25)	3252 (98.9%)	302 (53.7%)
	[25, 50)	31 (0.9%)	96 (17.1%)
	[50, 75)	4 (0.1%)	68 (12.1%)
	[75, 100]	1 (<0.1%)	96 (17.1%)
At month 3 Day 60 to 90	Positive/negative	144 (6.0%)/2276 (94.0%)	204 (50.5%)/200 (49.5%)
	Retained	2420 (73.6%)	404 (71.9%)
At Month 6 Day 150 to 180	Positive/negative	109 (5.6%)/1850 (94.4%)	128 (40.9%)/185 (59.1%)
	Retained	1959 (59.6%)	313 (55.7%)
At Month 9 Day 240 to 270	Positive/negative	88 (5.2%)/1601 (94.8%)	104 (39.0%)/163 (61.0%)
	Retained	1689 (51.4%)	267 (47.5%)
At Month 12 Day 330 to 360	Positive/negative	80 (5.3%)/1443 (94.7%)	74 (31.2%)/163 (68.8%)
	Retained	1523 (46.3%)	237 (42.2%)
Retained/not retained 365 Days		1447 (44.0%)/1841 (56.0%)	224 (39.9%)/338 (60.1%)

Descriptive statistics were summarized for baseline characteristics of patients, and standardized differences were used to compare patient groups. Patients were considered baseline BZD users if they had any BZD positive urine samples in the first month of treatment

## Results

### Patient demographics

Our cohort consisted of 3850 patients, with a median age of 31.4 years old and 60% of patients being male. 36% of patients resided in Northern Ontario and 16% lived in a rural setting. The Cox proportional hazard model found that those patients living in the North were 40.7% less likely to drop out of treatment by the one-year mark compared to patients in the South. Male patients were 30.2% more likely to drop out of treatment than female patients. There were no significant differences in treatment retention for patients living in rural or urban centres.

### Baseline BZD use

Patients were stratified by baseline BZD use, which was defined by the presence of any BZD-positive urine samples in the first month of treatment. Of the 3850 patients, 562 (15%) were considered baseline BZD users and 3288 (85%) non-users. The ratio of female to male patients was greater in the BZD use group with 43.8% of BZD users being female, compared to only 39.9% of non-users being female. Female patients were 34.5% more likely to be baseline BZD users than were males, according to the Cox proportional hazard model. Another difference in the two groups was in age, with the positive group having a greater median age of 34.3 years compared to 30.8 years. Hazard ratios found that patients were 25.5% less likely to have a BZD-positive urine sample in their first month of treatment if they lived in a rural area and were 23.6% less likely to have a BZD-positive urine sample in their first month of treatment if they lived in the North. Compared to patients who were considered non-baseline users, baseline BZD users had an increased median peak dose of methadone (85 vs. 75 mg) and had a lower median retention of 215 days, compared to 265 days.

### Retention and baseline BZD use

The following variables were included in the Cox proportional hazard model: age [_a_HR = 0.98 (95% CI 0.975–0.984)], gender (female [_a_HR = 0.768 (95% CI 0.70–0.84)]), geography (North [_a_HR = 0.59 (95% CI 0.538–0.655)] and rural [_a_HR = 0.982 (95% CI 0.863–1.118)], and first-month BZD use [_a_HR = 1.149 (95% CI 1.022–1.292)]. For those patients considered baseline BZD users, the one-year retention rate was 39.9%. For those patients who were BZD negative on admission, the one-year retention rate was 44%. Of the first month BZD users that remained at one year, 31.2% were BZD positive. Of the 562 patients who were BZD positive in their first month of treatment, 49% of those that were retained were negative at 3 months, 59% at 6 months, 61% at 9 months, and 69% at the one-year mark. Of the baseline non-users who remained at the one-year mark, 95% were BZD negative. Importantly, patients were 14.9% more likely to drop out of treatment if they had BZD-positive urine samples in their first month of treatment.

### Proportion of BZD-positive urine samples

**Fig. 2:**
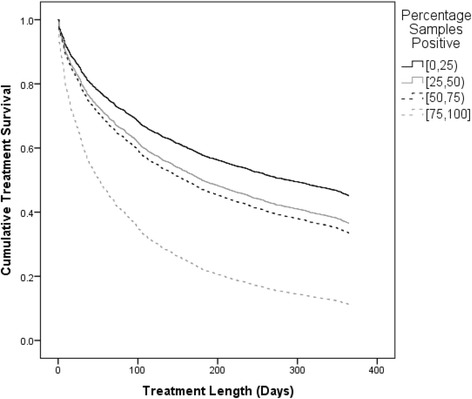
Treatment retention by proportion of BZD-positive urine samples. A Cox proportional hazard analysis was used to characterize the time to treatment discontinuation across the four patient groups. Patients with 25–50% of urines BZD-positive were 26.6% more likely to drop out of treatment than patients in the 0–25% reference group [_a_HR = 1.26 (95% CI 1.02–1.57)]. Patients with 50–75% of urines BZD-positive were 37.4% more likely to drop out of treatment than patients in the 0–25% reference group [_a_HR = 1.37(95% CI 1.03–1.832)]. Patients with 75–100% of urines BZD positive were 174.4% more likely to drop out of treatment than patients in the 0–25% reference group [_a_HR = 2.74(95% CI 2.19–3.43)]

In addition to being categorized by first-month BZD use, patients were also stratified by the proportion of BZD-positive urine samples throughout treatment: 0–25, 25–50, 50–75, and 75–100%. Of the 3850 patients, 3556 had BZD-positive urine samples less than 25% of the time. These patients experienced a one-year retention rate of 45%. 127 patients had BZD-positive urine samples 25–50% of the time, with a retention rate of 32%. These patients were 26.6% more likely to not be retained, compared to those patients in the <25% group. 72 patients had BZD-positive urine samples between 50–75% of the time, and they experienced a retention rate of 33%. These patients were 37.4% more likely to terminate treatment prematurely than the <25% group. Lastly, 97 patients had BZD-positive urine samples more than 75% of the time, and they suffered the lowest retention rate of 14%. These patients were a substantial 174.4% more likely to not be retained in treatment, compared to the <25% group (Fig. 2).

### BZD use and geography

**Fig. 3:**
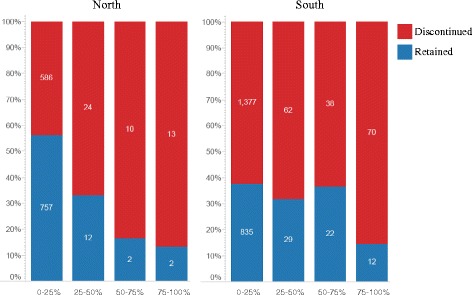
Proportion of patients retained in treatment for one year, by frequency of BZD use, comparing North vs. South. The pattern of decreased retention with increased proportion of BZD-positive urine samples is not seen in the Southern population, as it is in the Northern population. However, Southern patients with a high proportion of BZD-positive urine samples (≥75%) experienced decreased retention rates

Patients were categorized as residing in Northern Ontario or Southern Ontario according to the Local Health Integration Network (LHIN) in which they lived; patients residing in LHIN 13 or 14 were considered Northern residents. For the proportion of BZD-positive urine samples, patients in the North had retention rates of 56, 33, 17, and 13%. Patients in the South experienced retention rates of 38, 32, 36, and 14%. In the Northern population, the greater the proportion of BZD-positive urine samples, the lower the retention; however, in the Southern population, the decrease was less pronounced (Fig. 3). Instead, it appears that only patients with >75% of urines BZD positive suffer the lowest retention rates.

## Discussion

Previous studies have revealed mixed findings about whether BZD use impacts OAT retention. A review of BZD use in OAT suggests that most studies have found that baseline BZD use is not predictive of decreased retention [6]; however, this is contrary to our findings. Our findings support the idea that baseline BZD use is predictive of treatment drop out, with these patients being 14.9% more likely to terminate treatment prematurely. Additionally, our results indicate that with increasing proportion of BZD-positive urine samples, patients are at increased risk of premature drop out; therefore, both BZD use at treatment outset and intensity of BZD use during OAT are correlated with decreased retention. Patients taking BZD may be more likely to drop out of treatment given that BZD use is associated with polydrug use, risk-taking behaviors, unemployment [22, 23], involvement in criminal activity [23], and psychological distress [10]. These additional complexities may hinder patients’ ability to thrive in treatment and make them more likely to terminate treatment prematurely. Additionally, patients using BZD while receiving OAT are at increased risk of overdose and death [1, 24], an event that would be captured as premature drop out in our analysis.

Compared to previous studies on patients in OAT, the median age of our patient sample was younger, at 31 years (median age = 34.6 [10]; median age = 35 [9]; median age = 47 [25]); however, the age distribution in our dataset seems to reflect the opioid dependent population in Ontario at the time of the study. This is supported by a cross-sectional study of all opioid-related deaths in Ontario between 1991 and 2010, which found that most opioid-related deaths occurred among young adults aged 25–34 [2]. A study relying on self-reports of high school students found that opioid use is increasing among adolescence [26]; therefore, it is possible that the opioid-dependent population in Ontario has gotten younger in recent years, compared to the times and locations at which the other studies on BZD use were conducted.

In terms of concurrent drug use, 15% of our patient population was BZD-positive in their first month of treatment. Compared to previous studies, this number is low [6]. A review by Lintzeris et al. found that problematic BZD use amongst patients receiving OAT varies from 18 to 50% [6]; however, it is important to consider that many of these studies are international, as drug consumption and treatment differ throughout the world. At the time of this study, methadone and buprenorphine/naloxone were the only two approved medications for OAT in Canada; however, in other parts of the world, other forms of OAT—including heroin assisted therapy, slow-release morphine, and intramuscular slow-release naltrexone—exist, which have been shown to be effective in the treatment of opioid dependence [27, 28]. Regarding BZD use among patients in OAT, studies have found that BZD use is common not only in Canada, but also in countries across Europe, North America, and in Australia [8, 29–31].

Our findings indicate that BZD use is more prevalent in the female population, which is supported by previous studies [10]. The higher prevalence of BZD use in this population may be explained by the fact that females in OAT suffer from more psychiatric comorbidity than their male counterparts [32, 33]. Studies have also found that female patients are more likely to receive a BZD prescription than males, [29] and that the risks associated with prescribed BZD (e.g., hospital visits, accidental injury) are increased in the female population [34]. Given that previous studies have found that females are more likely to use BZD, more likely to be prescribed BZD, and more likely to suffer from psychological comorbidities, it would seem reasonable that females are also more likely to self-medicate with non-prescribed BZD as well.

We also found a difference in the age of baseline users and non-users, with baseline users having a median age of 34.3 years and non-users having a median age of 30.8 years. It may be the case that patients who have more severe mental health disorders (and use BZD to self-medicate) take longer to present to treatment.

Our results suggest that geography is an important factor to consider when studying BZD use. We found that patients living in rural areas, and those patients living in the North, were less likely to be baseline BZD users. When studying the impact of geography on retention, we confirmed the earlier finding that patients in the North were more likely to be retained in treatment; however, we failed to confirm the correlation between rurality and retention in this smaller sample size [18]. The higher rates of retention in the North may seem surprising, given that these patients face a variety of barriers when accessing health care. In remote Northern communities, patients often have to travel long distances to access OAT-prescribing physicians and the pharmacy that dispenses their methadone or buprenorphine [18]. It seems somewhat intuitive that given the added difficulties, these patients would experience decreased retention; however, this is not the case. In fact, patients in the North were 41% less likely to terminate treatment prematurely than were Southern patients. Given that Northern patients are less likely to be BZD users and BZD use is predictive of drop out, the higher rates of retention may be partially explained by less BZD use in the North. However, given that the Northern population was more likely to use cocaine and that cocaine use is predictive of treatment drop out [20], there are likely other reasons why this population benefits from greater retention. Northern patients who overcome the barriers to treatment entry may be more motivated to be successful in their treatment. The decreased BZD use in Northern and rural patients may be explained by inaccessibility in terms of availability or cost of non-prescribed BZD. Although we were unable to quantify illicit BZD use, we would expect that illicit BZD would be less accessible in Northern Ontario than other drugs, including cocaine and non-prescribed opioids.

Of the 3288 patients who were considered baseline non-users, the vast majority (~95%) of patients who were retained were also BZD negative at the one-year mark. For the remaining 5%, it may be the case that patients received a BZD prescription during the course of their treatment. Of the 562 patients who were BZD-positive in their first month of treatment, the proportion that continued to have BZD-positive samples declined over time retained in treatment. Given that OAT does not treat BZD dependence, it may seem surprising that the majority of BZD users terminate BZD use during treatment; however, it may be the case that many of the initially positive patients were heavy BZD users and dropped out of treatment before reaching the 3rd, 9th, or 12th month. It may also be the result of contingency management, whereby a patient is motivated to abstain from concurrent drug use in order to obtain carried (i.e., take home) doses. Contingency management might encourage a patient to decrease BZD use, given that it has been shown to reduce the use of other drugs in patients who are receiving OAT [21].

Although our patient sample had fewer BZD users than expected, it is important to consider why BZD use is so prevalent in OAT. One theory is that there is an increased prevalence of comorbid mental health issues—including depression, anxiety, and insomnia—among patients enrolled in OAT [35, 36]. It is for this reason that patients are often prescribed BZD to treat their concurrent mental health disorders [37]. A retrospective cohort study of over 2000 patients receiving OAT found that 40% of patients had received at least one BZD prescription [29]. The current guidelines on how to manage patients with opioid dependence and co-occurring mental health disorders—for which BZD are clinically indicated—are unclear [38]. Further research needs to inform physicians as to how they should manage these complex patients. However, it is important to note that not all BZD use is prescribed. A retrospective study of patients receiving methadone found that nearly 35% of patients who were BZD-positive did not have an associated prescription [39].

One of the main limitations of this study is the inability to detect whether a patient received a BZD prescription from a physician other than their OAT provider. The number of patients that received a BZD prescription from their OAT provider was known; however, this number was less than 7.2%. This number is likely this low due to the majority of BZD prescriptions coming from physicians outside the OATC network. A retrospective cohort study that utilized a prescription database found that over 60% of BZD prescriptions were written by a physician other than the patient’s OAT provider [29]; therefore, it is very likely that our detection of BZD prescriptions is insufficient. While this study captures the impact of general BZD use on OAT retention, it does not necessarily differentiate between clinical BZD use for mental health disorders and non-prescribed use. It is possible that a large proportion of our patient sample did have BZD prescriptions and were using BZD as clinically prescribed from a non-OATC physician. Another limitation is that we were unable to determine the dose of BZD taken by patients. Lastly, if a patient dropped out of treatment, we were not able to determine whether the patient simply terminated all OAT, began treatment at a non-OATC clinic, was incarcerated, hospitalized, or died.

This study also has several strengths. While BZD use in OAT has been studied previously, it has not been studied in a regional context—in this case Northern vs. Southern Ontario. Of the studies that have been done, few have been longitudinal. Those studies that were longitudinal either had a much smaller sample size, relied on self-reported data, or did not use a survival analysis to quantify the impact of BZD use and geography on treatment retention. Other strengths regarding study design include the large sample size, the method of data collection, and the patient population. With nearly 4000 patients in our cohort, this study captures a substantial proportion of all patients receiving OAT in Ontario. Additionally, this study did not rely on patient self-report, as did many of the previous studies. Lastly, the fact that all patients in the cohort were from one network of clinics adds strength to the comparisons made between patients.

## Conclusions

The findings of this study suggest that BZD use is a marker for a greater clinical complexity, and puts patients at increased risk of premature OAT discontinuation. Given that treatment retention is correlated with better patient outcomes [40, 41], it is important that physicians be cognizant of BZD detection in patients’ urine samples, as this could be a marker for decreased retention. Additionally, physicians should exercise caution when prescribing BZD to patients who are receiving OAT; however, further research needs to be done to better understand how prescribed BZD use and non-prescribed use differentially impact treatment retention. Employing mixed methodologies to understand factors such as reason for BZD use or reason for treatment termination would also add strength to this study.

## Abbreviations

BZD: Benzodiazepines
LHIN: Local Health Integration Network
OAT: Opioid agonist therapy
OATC: Ontario addiction treatment centres.
