# Erratum to: The impact of benzodiazepine use in patients enrolled in opioid agonist therapy in Northern and rural Ontario

**DOI:** 10.1186/s12954-017-0140-7

**Published:** 2017-03-28

**Authors:** Alexandra M. Franklyn, Joseph K. Eibl, Graham Gauthier, David Pellegrini, Nancy E. Lightfoot, David C. Marsh

**Affiliations:** 1 Northern Ontario School of Medicine, Sudbury, ON P3E 2C6 Canada; 2 School of Rural and Northern Health, Laurentian University, 935 Ramsey Lake Rd, Sudbury, ON P3E 2C6 Canada; 3 Canadian Addiction Treatment Centers, 13291 Yonge St., Ste. 403, Richmond Hill, ON L4E 4L6 Canada

## Erratum

After publication of the original article [1], it was noted that one of the author’s name and affiliation was presented incorrectly. The correct name of the author (Nancy E. Lightfoot) and affiliation (^2^School of Rural and Northern Health, Laurentian University, 935 Ramsey Lake Rd., Sudbury, ON P3E 2C6, Canada) is published in this Erratum for quick reference. The original article was corrected. We apologise for any confusion this may have caused.
